# Patterns of multimorbidity and their association with health outcomes within Yorkshire, England: baseline results from the Yorkshire Health Study

**DOI:** 10.1186/s12889-016-3335-z

**Published:** 2016-07-27

**Authors:** Jessica Li, Mark Green, Ben Kearns, Eleanor Holding, Christine Smith, Annette Haywood, Cindy Cooper, Mark Strong, Clare Relton

**Affiliations:** 1 School of Health and Related Research (ScHARR), University of Sheffield, Sheffield, UK; 2 Department of Geography & Planning, University of Liverpool, Liverpool, UK; 3 Barnsley Hospital NHS Foundation Trust, Barnsley, UK; 4 Public Health Section, ScHARR, Regent Court, University of Sheffield, 30 Regent Street, Sheffield, S1 4DA UK

**Keywords:** Multimorbidity, Multiple cohort trial, Health conditions, Long-term conditions

## Abstract

**Background:**

Multimorbidity is increasingly being recognized as a serious public health concern. Research into its determinants, prevalence, and management is needed and as the risk of experiencing multiple chronic conditions increases over time, attention should be given to investigating the development of multimorbidity through prospective cohort design studies. Here we examine the baseline patterns of multimorbidity and their association with health outcomes for residents in Yorkshire, England using data from the Yorkshire Health Study.

**Methods:**

Baseline data from the Yorkshire Health Study (YHS) was collected from 27,806 patients recruited between 2010 and 2012. A two-stage sampling strategy was implemented which first involved recruiting 43 general practice surgeries and then having them consent to mailing invitations to their patients to complete postal or online questionnaires. The questionnaire collected information on chronic health conditions, demographics, health-related behaviours, healthcare and medication usage, and a range of other health related variables. Descriptive statistics (chi-square and t tests) were used to examine associations between these variables and multimorbidity.

**Results:**

In the YHS cohort, 10,332 participants (37.2 %) reported having at least two or more long-term health conditions (multimorbidity). Older age, BMI and deprivation were all positively associated with multimorbidity. Nearly half (45.7 %) of participants from the most deprived areas experienced multimorbidity. Based on the weighted sample, average health-related quality of life decreased with the number of health conditions reported; the mean EQ-5D score for participants with no conditions was 0.945 compared to 0.355 for participants with five or more. The mean number of medications used for those without multimorbidity was 1.81 (range 1-13, SD = 1.25) compared to 3.81 (range 1-14, SD = 2.44) for those with at least two long-term conditions and 7.47 (range 1-37, SD = 7.47) for those with 5+ conditions.

**Conclusion:**

Patterns of multimorbidity within the Yorkshire Health Study support research on multimorbidity within previous observational cross-sectional studies. The YHS provides both a facility for participant recruitment to intervention trials, and a large population-based longitudinal cohort for observational research. It is planned to continue to record chronic conditions and other health related behaviours in future waves which will be useful for examining determinants and trends in chronic disease and multimorbidity.

## Background

Within the past decade, there has been a growing interest in researching the management, needs, and treatment of those with multiple long term health conditions [1]. Living with two or more chronic conditions has increasingly become more common as people age, leading to increased financial pressures on healthcare systems and treatment burden for those living with multimorbidities. The needs of these patients are complex, with those experiencing different conditions needing to attend multiple appointments with various doctors, while managing several medications [2]. This can often lead to inefficiencies in health care services, with patients attending multiple appointments, and confusion for patients who may receive conflicting advice from different specialists involved in their care [2–4]. Multimorbidity has also been found to be to be associated with increased healthcare costs, mortality rates, service use, and decreased physical functioning and quality of life [2, 3, 5–7], highlighting the need for more research into the key determinants to detect early signs of risk factors and assess effective interventions.

Currently, multimorbidity is most commonly defined as the coexistence of two or more chronic conditions [5, 8, 9]. These conditions can range across different long-term, disorders, illnesses, and health problems [3, 8–10]. Quantifying multimorbidity proves to be challenging because there has yet to be an internationally recognised list of conditions defined as chronic [4]; one recent systematic review of observational studies on multimorbidity within primary care found that the number of conditions studied ranged from five to 335 [11]. While there has been considerable discussion around the terms multimorbidity and comorbidity (the occurrence of medical conditions additional to an index disease) [9, 12, 13], here we focus on multimorbidity. This is based on the argument that multimorbidities may be a better construct for primary care when the focus is on the individual as a whole compared to comorbidity which may be more useful in specialist care where the emphasis is on an index disease [8].

Most research on multimorbidity is dominated by its effect on individuals and healthcare services, but the evidence base for complex interventions within primary care is very limited and little is known about patient needs [5, 14]. A recent Cochrane review found that most studies have focused on multimorbidity in older patients and evidence for interventions has been mixed, concluding that further research into particular risk factors and functional difficulties was needed [10]. There has also been little research into how patients experiencing multimorbidity view their condition, their experiences with health services and providers, and how this relates to the professional construction of multimorbidity [5, 8, 11]. Qualitative work among older patients with diabetes, depression, and osteoarthritis found that this group wanted convenient access to health care, individualised care plans, support from one coordinator of care, and continuity of relationships with health professionals [15], stressing the complex needs of those with multimorbidities.

Research into multimorbidity is fairly recent, and the epidemiology of multimorbidity is not well understood, but is required to help inform decision-making. The aim of this study was to use baseline data from the Yorkshire Health Study (YHS) to examine the patterns of multimorbidity and their association with health outcomes for residents in Yorkshire, England.

## Methods

### Data, recruitment, sampling

The Yorkshire Health Study is a longitudinal panel study that collects health information on adults aged 16-85. It was originally designed to address the needs of the Department of Health *Healthy Weight, Healthy Lives* report [16], which could not be addressed by existing population cohorts. The first wave of data was collected between 2010 and 2012 and surveyed patients residing in Yorkshire, England. Invitations to complete postal or online questionnaires were distributed to 156,866 patients (aged 16-85) recruited from 43 general medical practices of which 27,806 questionnaires were returned (15.9 % response rate). The YHS has a small undercount of younger males and those from deprived areas. Further details on the data collection methods used and how the cohort demographic profile compares to the general Yorkshire population have been published elsewhere [17, 18] and the second wave of data collection was completed in January 2016.

A unique design feature of the cohort is that it is the first cohort study to use the ‘cohort multiple randomised controlled trial design’ [19], an innovative approach to tackling some of the problems associated with pragmatic trial designs (recruitment, ethics, patient preferences and treatment comparisons). The design enables quick and easy identification of participants to multiple randomized controlled trials within the same population, facilitating easier comparability between studies. For each randomised controlled trial, those eligible are identified and a random selection offered the trial intervention; their outcomes are compared with those eligible patients not offered the intervention.

### Measures

One of the original aims for the cohort was to initiate a programme of research into the management and self-management of weight and long-term conditions in adults living in the South Yorkshire area [18]. Because of this, a wide range of information was collected allowing for detailed investigation of variations in obesity in relation to other dimensions of health, particularly chronic health conditions. Respondents were able to report on twelve ‘long standing’ conditions listed in the questionnaire: tiredness/fatigue, pain, insomnia, anxiety/nerves, depression, diabetes, breathing problems, high blood pressure, heart disease, osteoarthritis, stroke, and cancer. An open ended option was also provided which allowed respondents to report any other conditions not listed.

Demographic information was collected on sex, age, self-reported height, weight, and waist measurement (tape measure was provided), ethnicity, highest level of education, occupation (using 2010 National Statistics Socioeconomic Classification), and postcode information was used to measure deprivation via the 2010 Index of Multiple Deprivation (IMD). The IMD is an area-based measure for multiple deprivation for small areas in England [20]. 2010 IMD scores calculated based on 38 indicators across seven domains (e.g. income, employment, health, education) range from 0.53 to 87.80 (higher values indicating higher deprivation) and are assigned to 32,482 areas in England (also known as Lower layer Super Output Areas). IMD scores were categorised into quintile groups for our analyses. Health information related to frequency of health care usage, medication usage (non-prescription and prescription), and health-related quality of life using EuroQoL-5D (EQ-5D-3L) were also collected. The EuroQoL is a standardised instrument used for measuring five attributes of health status: mobility, self-care, usual activities, pain/discomfort, and anxiety/depression [21]. The full original questionnaire and details about the distributions of these variables have been published [17, 18].

### Analyses

Data was analysed using SPSS Windows v22.0. Descriptive statistics of baseline data are presented in tables, cross-tabulations, and graphical displays to illustrate patterns of multimorbidity across demographics, health-related quality of life, health service, and medication usage. Multimorbidity was defined as those who self-reported having at least two of the thirteen long-term health conditions (i.e. included those responding with an ‘other’ condition) listed in the questionnaire. Bivariate analyses were conducted to determine associations between multimorbidity and demographics, deprivation, EQ-5D scores, health service use and medication usage. A t-test was used to analyse differences in mean number of long term health conditions across gender and ethnicity and one-way ANOVA was used for differences across age groups, deprivation quintile groups, and body mass index (BMI) groups. Chi-squared tests were used to measure differences in the prevalence of multimorbidity between all demographic variables (p < 0.05). Sample weights were applied to data to adjust for groups who were over-represented in the cohort compared to the general adult Yorkshire population (females, those who are older and those living in less deprived areas) [17, 22]. With the exception of Table 1, data presented here are based on the weighted sample.

**Table 1:** Unweighted sample characteristics and long standing health conditions for Yorkshire Health Study Wave 1 (*N* = 27,806)

	n (%)	Mean number of conditions (SD^a^)^b^	2 or more conditions^c^: n (%)
Gender			
Male	12155 (43.7)	1.50 (1.78)	4551 (37.4)
Female	15651 (56.3)	1.49 (1.80)	5781 (36.9)
Age Group			
≤24	1734 (6.2)	0.47 (0.92)	188 (10.8)
25-34	2639 (9.5)	0.63 (1.15)	391 (14.8)
35-44	3516 (12.6)	0.83 (1.35)	708 (20.1)
45-54	4489 (16.1)	1.19 (1.62)	1302 (29.0)
55-64	5938 (21.4)	1.63 (1.80)	2412 (40.6)
65-74	5827 (21.0)	2.09 (1.93)	3068 (52.7)
≥75	3254 (11.7)	2.53 (1.93)	2125 (65.3)
Ethnicity			
White	26419 (95.0)	1.51 (1.80)	9961 (37.7)
Non-White	1095 (3.9)	1.05 (1.61)	278 (25.4)
Deprivation Quintile			
Least Deprived	3948 (14.2)	1.08 (1.46)	1060 (26.8)
Q2	6923 (24.9)	1.30 (1.62)	2275 (32.9)
Q3	4602 (16.6)	1.39 (1.69)	1615 (35.1)
Q4	5116 (18.4)	1.62 (1.85)	2098 (41.0)
Most Deprived	7142 (25.7)	1.88 (2.03)	3263 (45.7)
Body Mass Index^d^			
Underweight	436 (1.6)	1.29 (1.88)	126 (28.9)
Normal	11101 (39.9)	1.10 (1.51)	3040 (27.4)
Overweight	9671 (34.8)	1.52 (1.74)	3772 (39.0)
Obese	5190 (18.7)	2.26 (2.10)	2833 (54.6)

Missing data not shown in table

^a^ Standard deviation

^b^ Differences between means within ethnicity, age-group, deprivation and BMI group differed significantly p < 0.001 (t test for independent samples for ethnicity; one-way ANOVA for age-group, deprivation, and BMI group)

^c^ Differences between categories within age, ethnicity, deprivation, and BMI group differed significantly p < .0001 (χ^2^ test for 2 × n tables)

^d^ Calculated by self-reported weight (kg) divided by height-squared (m^2^). Categories are based on WHO cut-offs

## Results

**Fig. 1:**
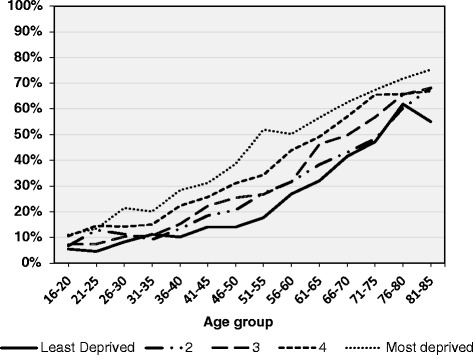
Multimorbidity among Yorkshire Health Study participants

Table 1 presents sample characteristics, mean number of long standing conditions, and proportions of those with multimorbidity. Within the unweighted sample, slightly over one-third (*n* = 10,332) had experienced multimorbidity (reported having two or more long-standing conditions). This estimate lowered to 31 % when based on weighted data (data not shown). The mean number of self-reported long-term health conditions varied significantly depending on age, ethnicity, index of multiple deprivation (IMD) quintile and BMI group. Multimorbidity was higher among those who were older, of white ethnicity, resided in more deprived areas and were overweight or obese. Overall, 65 % of those aged 75+, 38 % of white participants, and 55 % of obese participants reported having at least two long-term health conditions. These demographic associations remained statistically significant when analyses were conducted on weighted data (results not presented), and also indicated a significant difference in multimorbidity between males and females (29 % vs. 32 % respectively). Within the cohort, there was a clear social gradient between deprivation and multimorbidity. Nearly half (46 %) of those living in the most deprived areas experienced multimorbidity compared to 27 % of those living in the least deprived areas (Table 1). This link between high deprivation and multimorbidity was consistent across all age groups, with prevalence reaching the highest for those aged over 80 years and living in the most deprived areas (over 70 %) (Fig. 1).

**Fig. 2:**
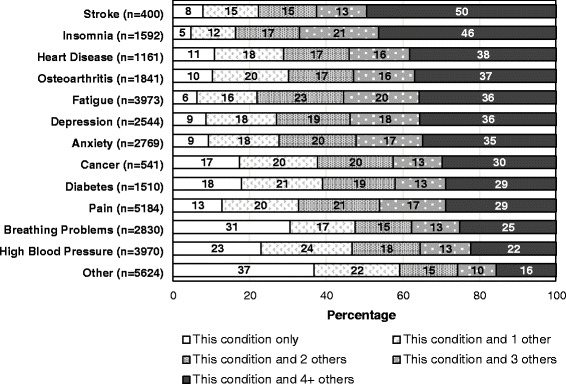
Distribution of long-standing conditions reported by Yorkshire Health Study participants

**Fig. 3:**
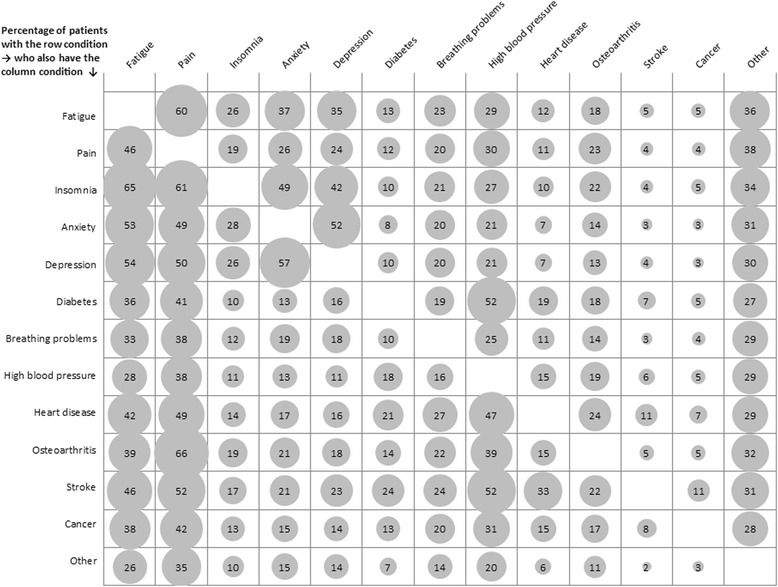
Self-reported long-term health conditions among Yorkshire Health Study participants

Of those who reported any long term-conditions (*n* = 16,760), 38.4 % (*n* = 6428) reported experiencing only one condition. Those who reported a stroke (*n* = 400) reported experiencing more multiple long-term limiting conditions, with 50 % reporting experiencing four or more additional chronic conditions (Fig. 2). Insomnia was the second highest condition with 46 % of those experiencing this condition along with at least four others. With the exception of ‘other’ conditions, the condition with the least frequently reported number of conditions alongside it was breathing problems, 31 % of these participants only experienced it alone. Figure 3 specifies the proportion of accompanying conditions alongside each condition. Roughly half of those who had reported stroke also reported experiencing fatigue, chronic pain, and high blood pressure. Osteoarthritis and pain was the most frequent combination of two conditions (66 % of those with arthritis reported also having pain), followed by insomnia and fatigue (65 % of those with insomnia reported fatigue). Fatigue and pain were commonly reported among other long-term health conditions (Fig. 3).

**Fig. 4:**
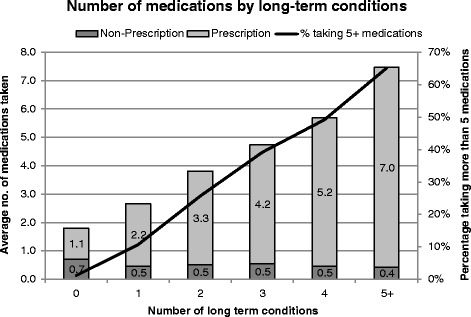
Yorkshire Health Study medication use by long-term conditions

Within the cohort there was a negative relationship between number of self-reported long-term conditions and self-reported quality of life (Table 2); Participants with five or more conditions reported a mean EQ-5D score of 0.355 (range -0.594-1, SE = 0.008), nearly three times lower than the mean score for participants with no conditions 0.945 (range -0.594-1, SE = 0.001). Multimorbidity was positively associated with more health service visits (Table 3) and medication use (Fig. 4). Participants with no long term health conditions reported taking on average 1.81 medications (non-prescription and prescription) compared to those with at least two long-term conditions who reported 3.8 medications and those with 5+ conditions who reported 7.5 medications on average.

**Table 2:** EQ-5D by number of long-term conditions

Number of conditions	Mean^a^	Standard error	95 % confidence interval	N
0	0.945	0.001	0.943 – 0.947	12978
1	0.859	0.002	0.854 – 0.863	6012
2	0.754	0.004	0.747 – 0.762	3279
3	0.651	0.006	0.640 – 0.662	2083
4	0.532	0.008	0.516 – 0.549	1297
5+	0.355	0.008	0.339 – 0.371	1677

^a^ Range = 1 (perfect health) through to 0 (dead) to -0.594 (worse than dead) [31]

EQ-5D score not available for all participants

**Table 3:** Healthcare use in last three months by long-term conditions (mean number of appointments, standard error)

Number of LTCs^a^	Inpatients	Outpatients	Day cases	A&E^b^
0	0.11 (0.010)	0.26 (0.011)	0.07 (0.009)	0.11 (0.005)
1	0.29 (0.041)	0.72 (0.031)	0.16 (0.015)	0.16 (0.009)
2	0.52 (0.063)	1.09 (0.049)	0.33 (0.051)	0.23 (0.015)
3	1.10 (0.218)	1.35 (0.073)	0.44 (0.073)	0.35 (0.034)
4	1.13 (0.190)	1.76 (0.127)	0.49 (0.070)	0.46 (0.053)
5+	2.05 (0.278)	2.51 (0.138)	1.10 (0.171)	0.59 (0.049)

^a^ LTCs = Long-term conditions

^b^ Accident and Emergency

## Discussion

Research into multimorbidity has been steadily increasing over the years. Therefore a standard definition of multimorbidity is required if research in this area is to advance. The level of prevalence of multimorbidity can vary across studies depending on the measure used (e.g. prevalence estimates have ranged from 13 to 95 % across primary care studies in Europe, North America and Australia [11]). Conditions within multimorbidity vary from study to study [5, 11, 23]. For instance, some might classify depression and anxiety separately while others could argue that because they are clinically closely inter-related they should not be treated as separate entities when classifying those with multimorbidity [6, 8]. In this study we chose to examine depression and anxiety as separate entities when estimating multimorbidity as has been done elsewhere [3]. Based on a simple multimorbidity definition (the co-occurrence of two or more conditions whether coincidental or not [12]), overall we found that 37 % of participants sampled in the Yorkshire Health Study had experienced multimorbidity. This lies in between other UK estimates of multimorbidity that have ranged from 23 % [3] and 30 % in Scotland [24] to 58 % in England [4]. However it is important to bear in mind that each study used a different range of long-term conditions when estimating multimorbidity (e.g. learning disability, thyroid disease, epilepsy, etc.). Within the weighted sample, females were also more likely to report experiencing multimorbidity, consistent with other UK, European, and American studies [1, 11, 25]. Consistent with trends [3], multimorbidity steadily increased with age in the YHS cohort. A clear link between multimorbidity and deprivation within the YHS cohort was also identified, supporting evidence found elsewhere which indicate higher levels of multimorbidity among lower socioeconomic groups [3, 4].

Regarding health outcomes associated with multimorbidity, our findings suggest that multimorbidity is associated with poor health-related quality of life (measured through EQ-5D scores), which is consistent with findings from previous studies that have used other health-related quality of life questionnaires (such as the SF-36) [6]. Patterns of increasing recent hospital, GP, nurse, dietician, physiotherapist, alternative therapist, and other carer visits among those with more long-term conditions were also identified. Health service use in future waves of the YHS can be compared to multimorbid participants’ baseline service use and used to prioritize and adapt the commissioning and management of future services, particularly by identification of need in different populations and locations (e.g. areas of high deprivation). Guidance on the decisions for multiple medication use are currently lacking within the United Kingdom [26] and findings from our study suggest that while the proportion of non-prescription and prescription medications taken is fairly consistent across the number of conditions reported, among YHS participants, the number of current medications increases substantially to an average of 7 medications for those with five or more self-reported long-term health conditions. Furthermore data from our survey supports the link between obesity and multimorbidity which has been found elsewhere [27]. Information on a number of weight management strategies within the YHS [22] could be used to further explore this link and determine whether weight loss and strategies used would be associated with a reduction in the number of chronic conditions over time.

Some limitations of our data should be considered. Data within the YHS cohort is based on self-report, which is prone to known biases [28]. However, with regard to multimorbidity, a benefit of using self-report data is that it includes conditions that patients may not always report to clinicians or clinicians may under-record (e.g. anxiety or pain) [3]. Another limitation is that those who responded with an ‘other’ condition could have also responded with multiple conditions in the free text response (e.g. arthritis, hypothyroidism, high cholesterol, epilepsy, glaucoma, irritable bowel syndrome, etc.). Within this study we have not analysed these free text conditions, therefore the number of conditions for this category may be under or over estimated. Finally, though cross-sectional designs can provide prevalence estimates and descriptive information that would allow for identification of potential risk factors, they do not allow for a deeper understanding of patients’ experiences of living with multimorbidities over the life course and how these needs and experiences change as new morbidities are acquired over time. Because of this, prospective cohort studies have been regarded as the ‘gold standard’ for studying multimorbidity [5]. One recent systematic review of six prospective cohort studies within primary care found that these studies mainly focused on only healthcare utilisation, patient’s physical functioning, and risk factors and concluded that future cohort studies should also consider examining longitudinal links between socioeconomic factors and mental illness [5].

The National Institute for Health Care and Excellence (NICE) is currently consulting on clinical practice guidance for the management of multimorbidity, aiming to publish guidelines in 2016 [29]. Studies have indicated that more research should investigate modifiable risk factors (e.g. smoking and diet), socio-economic disparities in multimorbidity, and seek to examine multimorbidity longitudinally through large-scale prospective designs [5, 11]. Though not examined in this study, the second wave of data collection (2013-2015) for the YHS which was recently completed in January 2016 could provide further insight into addressing socioeconomic patterns of multimorbidity over the life course or other issues e.g. determining the average onset age for multimorbidity, identifying risk factors associated with multimorbidity over time (via health behaviours collected in the questionnaire but not examined in this study e.g. alcohol consumption, diet, and exercise), or assessing potential complex interventions within primary settings for patients experiencing multimorbidity and examining the cost-effectiveness of those interventions (through quality of life or life satisfaction measures). Because the YHS was designed to provide a cohort facility for multiple trials and other studies, its recruitment would allow researchers to examine specific subgroups (e.g. demographic) or groups that may be particularly vulnerable to multimorbidity (e.g. certain ethnic groups, patients experiencing specific conditions). The majority of the cohort (approximately 78 %) provided consent for researchers to access their health records [17] which would also enable data linkage with other disease diagnoses or healthcare and medication usage reported in other records. An ongoing example is a study that links YHS data with data from cancer screening programmes to examine the impact of morbidities (and hence multimorbidity) on the uptake of colorectal cancer screening [30].

## Conclusions

Within our baseline, our findings support evidence around poor health outcomes for those with multimorbidity and indicate a clear socio-economic patterning of multimorbidity, with those living in the most depriving areas being more likely to experience multimorbidities. Overall the YHS is a useful resource for those who are interested in chronic disease and multimorbidity research and would provide researchers with an efficient way of recruiting patients from a population-based cohort for a variety of different studies (e.g. randomised control trials, qualitative, cross-sectional, or longitudinal) alongside a dataset of rich information on a wide range of relevant health-related behaviours and conditions for analysing patterns and trends in chronic disease over time.

## Abbreviations

BMI, Body Mass Index; GP, General Practitioner; NHS, National Health Service; YHS, Yorkshire Health Study
